# A quick overview on some aspects of endocrinological and therapeutic effects of *Berberis vulgaris L.*

**Published:** 2015

**Authors:** Ali Zarei, Saeed Changizi-Ashtiyani, Soheila Taheri, Majid Ramezani

**Affiliations:** 1 *Young Researchers and Elite Club, Abadeh Branch, Islamic Azad University, Abadeh, Iran*; 2 *Department of Physiology, Arak University of Medical Sciences, Arak, Iran*; 3 *Education Development Center, Arak University of Medical Sciences, Arak, Iran*; 4 *Osteoporosis Research Center, Endocrinology and Metabolism Clinical Sciences Institute, Tehran University of Medical Sciences, Tehran, Iran*

**Keywords:** *Liver*, *Cholesterol*, *Diabetes*, *Thyroid*, *Barberry*, *Berberis Vulgaris*

## Abstract

Many herbaceous plants contain compounds that have biological effects in addition to their medicinal properties. They have compounds with numerous properties, including hypo lipidemic, hypoglycemic, antioxidant, and hepato protective ones, which have been analyzed at different levels. One of these plants, with the scientific name of *Berberis vulgaris*, is barberry. The most important compounds identified in this plant are berberine, oxycontin, palmatine, bervulcine, berbamine, columbamine, jatrorrhizine, coptisine, and berbamine. In addition to alkaloids, organic acids such as chelidonic acid, citric acid, malic acid, resin, tannin, pectinic, and mucilagic substances are among the ingredients of barberry.

In this paper, it was attempted to determine the role and effect of the extract of barberry on various body organs. The results showed that berberine actually increases insulin sensitivity and is capable of inhibiting alpha glucosidase, adipogenesis, and thus acts as an anti-obesity and hypoglycemic agent. Berberine reduces the density of serum cholesterol and triglycerides and can improve the function of liver enzymes, therefore, it can be suggested as a hypo lipidemic and hepato protective plant extract. The hepato protective effects of this extract are probably due to its antioxidant properties.

Studies showed that barberry have numerous health benefits, including anti-inflammatory ones. Moreover, it can be used as a medicinal herb to treat a variety of disorders, such as diabetes, liver disease, gallbladder pain, digestive, urinary tract diseases, and gallstones. However, more studies on this issue and doing more focused and intensive researches in this field are recommended.

## Introduction

Due to the multiple hazards of chemical compounds such as drug resistance, environmental problems, high production costs, and side effects, the tendency toward using medicinal plants in all health-related sciences is increasing (Rajaian et al., 2002 ▶).

**Figure 1:**
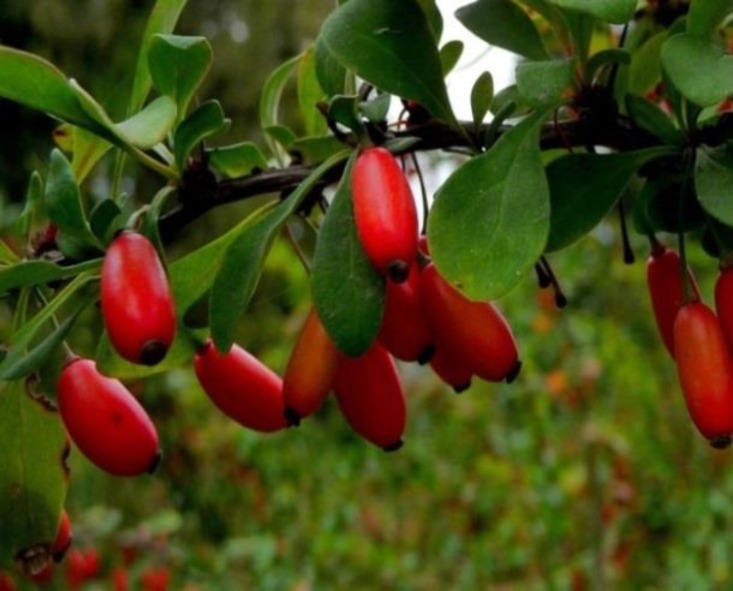
The aerial parts of *Berberis Vulgaris*

For centuries, medicinal plants have been the only available source for treating pains and pacifying human sufferings. In the present era, despite remarkable progress in the production and use of synthetic drugs, medicinal plants in all their pharmaceutical forms are still widely used. Iran, due to its climate variability, has rich vegetation, especially medicinal plants and consequently has had a long history in traditional medicine, a fact which makes it so reasonable to pay special attention to these invaluable resources (Changizi-Ashtiyani et al., 2013a ▶), (Figure 1).

In this regard, one of the medicinal plants is barberry, with the scientific name of *Berberis vulgaris *from the family of *Berberidaceae* (Figure 1) which grows abundantly in the mountainous north-east of Iran and Khorasan (Fatehi et al., 2005a ▶). The six known species of barberry include *Berberis crataegina, Berberis integerriam, Berberis khorasanica, Berberis orthobotrys, Berberis vulgaris, and Berberis thunbergii var. atropurpurea* (Fatehi et al., 2005b ▶).

Barberry is one of the plants that contain berberine and has had a long history in eastern and western medical tradition (Abd El-Wahab et al., 2013 ▶, Chevallier et al., 2001 ▶). In Iran and India, it goes back to at least 3,000 years (Souri et al., 2004 ▶, Timothy et al., 1997 ▶). According to traditional medicine, the fruit of this plant has a cold and dry nature. Ancient Egyptians used barberry and fennel seed to cure fever. Iranian physicians used it as a sedative and it has been used to treat diarrhea by Indian physicians (Chevallier et al., 2001 ▶, Kunwar et al., 2006 ▶).This plant is still used in northern Europe to treat disorders of the bladder, liver, and gallbladder. In Russia and Bulgaria, it is used to treat rheumatism, abnormal uterine bleedings (Fatehi et al.,2005 b ▶, Ivanovska et al.,1996 ▶), and in north America for malaria and general strengthening of the body (Imanshahidi et al., 2008 ▶). American Indians have used it as a medicine to help improve appetite (Abd El-Wahab et al., 2013 ▶). New experimental and animal studies have indicated the beneficial effects of this plant on blood pressure and lipid profiles, as well as its anti-inflammatory properties (Ebrahimi-Mamaghani et al., 2009 ▶).

Various parts of the plant including the bark, root, rhizomes, stem, leaf, and fruit are used in medicine (Arayne et al., 2007 ▶). Phyto chemical and pharmacological studies have shown that all the various species of the genus *Berberis* have antimicrobial, antiemetic, antipyretic, antioxidant, anti-inflammatory, anti-arrhythmic, sedative, anti-cholinergic, cholagogic, anti-leishmaniasis, and anti-malaria properties (Table 1).

In this paper, while generally referring to substances and active ingredients in the plant (Table 1) and reviewing the known effects of the extract of *Berberis vulgaris *(**Table 2**), we have paid especial attention to the impact of these compounds on some tissues and organs. Berberine and berbamine are the most important compounds found in all barberry species. Phytochemical analyses of different species of this genus have detected alkaloids, tannins, phenolic compounds, sterols, and triterpenes in them, too (Arham et al., 2012 ▶, Mokhber-Dezfuli et al., 2014 ▶). Berberin is the main active alkaloid with atetrabenzyl-hydroxy quinoline structure that can be found in all parts of the plant, especially in the roots. Berberine value of the fruit is about 5.5–7.7%. Berberine is not toxic at the doses used in clinical studies and no cytotoxic or mutagenic effects or adverse reactions have been observed (Shidfaraet al., 2012 ▶).

**Table 1:** Chemical compounds of *Berberis vulgaris L.* (Imanshahidi et al., 2008 ▶).

**Compound **	**Type**	**Part of plant**
Acanthine	Isoquinoline alkaloid	Root
		Bark
		Root bark
		Stem bark
		Shoots
		Leaf
		
Aesculetin	Coumarin	Fruit
Ascorbic acid	Vitamin	Fruit
		Leaf
Bargustanine	Isoquinoline alkaloid	Root
Berbamine	Isoquinoline alkaloid	Bark
		Root
		Steam bark
		
Berberrubine	Isoquinoline alkaloid	Root
Berberine	Isoquinoline alkaloid	Root
		Root bark
		Bark
		Stem bark
		Root wood
		Flowers
		Stem
		Fruit
		Shoots
		
Berberrubine	Isoquinoline alkaloid	Bark
Beriambine	Isoquinoline alkaloid	Root
Bervulcine	Isoquinoline alkaloid	Fruit
Caffeic acid	Phenylpropanoid	Fruit
Carotene, beta:	Carotenoid	Fruit
Chlorogenic acid	Phenylpropanoid	Fruit
Chrysanthemin	Flavonoid	Root
Columbamine	Isoquinoline alkaloid	Bark
		Stem bark
		
Delphinidin-3-o-beta-d-glucosido	Flavonoid	Leaf
Glucan, alpha:	Carbohydrate	Leaf
Hyperoside	Flavonol	Fruit
		Leaf
		
Jatrorrhizine	Isoquinoline alkaloid	Root
		Root bark
		Bark
		Stem bark
		
Lambertine	Alkane to c4	Root
Magnoflorine	Isoquinoline alkaloid	Root bark
		Root
		Stem bark
		
Pectin	Carbohydrate	Fruit
Pelargonin	Flavonoid	Root
Petunidin-3-o-beta-d-glucoside	Flavonoid	Bark
Polysaccharide	Carbohydrate	Root bark
Quercetin	Flavonol	Fruit
Sucrose	Carbohydrate	
Tannin	Tannin	Fruit
Thaliemidine	Isoquinoline alkaloid	Fruit
Tocopherol, alpha:	Oxygen heterocycle	Fruit
Urosolic acid	Triterpene	Fruit
		Leaf
		Leaf
Vitamin K	Vitamin	Fruit
		Fruit
Vulvracine	Alkaloid-misc	
Xylan, beta	Carbohydrate	Leaf

The most important alkaloids identified in the roots of the plant are magno florine, berberine, oxycontin, berbamine, palmatine, bervulcine, berlambine, berbamine, columbamine, jateorhizine, and coptisine. In addition to alkaloids, there are also organic acids such as chelidonic acid, citric acid, malic acid, resin, tannin, pectinic, and mucilagic substances in the roots (Fatehi et al., 2005a ▶, Zarei et al., 2012 ▶). The fruit is sour and contains sugars such as dextrose and fructose, malic acid, pectin, gum, tartaric, and citric acids. The berberine found in barberry root has anticonvulsant, sedative, and diuretic effects. Recent studies have suggested that berberine has other beneficial biological effects including anti-inflammatory ones (Javadzadeh et al., 2012 ▶).

Berberine is effective in the prevention of coronary artery diseases and may decrease the total cholesterol and triglyceride levels. The main effect of berbamine is blocking calcium channels. This alkaloid showed peroxidation activities in experiments on lipid peroxidation of red blood cells and could exert anti-myocardial ischemia and anti-arrhythmia effects. Moreover, oxycanthine acted as a sympatholytic agent and a vasodilator (Kong et al., 2004 ▶, Fatehi et al., 2005).

The aim of this study was to review the beneficial effects of *Berberis vulgaris *extract on the liver, thyroid, immune system, adipose tissue, and the plasma concentration of some biochemical factors.

## Materials and Methods

In this review article the role of barberry extract in different body tissues with respect to the literature and numerous empirical articles on this subject was reviewed. To do so, using proper key words, a comprehensive electronic search was done in authentic data bases such as Science Direct, Pub Med, Google Scholar, Persian Electronic Scientific Information Database (SID), Iran medex, and Magiran as well as the available paper resources in Persian scientific journals mainlybetween2008-2014.

The following keyword was used: “*Berberis vulgaris*”. To obtain additional data, a manual search was performed using the reference lists of included articles.

**The effect of** ***Berberis vulgaris*** **extract on lipid profiles**

Hyperlipidemia refers to a group of heterogeneous disorders characterized by high levels of blood lipid profiles. Hyperlipidemia, regardless of its cause, is a major, yet manageable risk factor for coronary heart diseases. The treatment goals can be designed on the basis of absolute levels of lipid and the presence or absence of risk factors. Studies show that the therapies leading to effective reduction of low-density lipoprotein (LDL) levels are clearly effective in decreasing mortality in patients with coronary artery disease. 

Studies show that the risk of recurring cardiovascular attacks in high-risk patients has a significant relationship with the period of using statins, as a group of common cholesterol-lowering drugs, which act by inhibiting the hydroxyl methylglutaryl coenzyme A reductase (HMG-CoA reductase) (Mancini et al., 2011 ▶).

Evidence suggests that long-term consumption of high-dose statins involves some less severe side effects such as headache, nausea and vomiting, constipation, muscle pain, and many serious side effects such as liver damage, rhabdomyolysis, renal failure, and loss or damage to memory as well as being forbidden during pregnancy (Rubenfire et al., 2010 ▶).That is why the tendency toward using herbs as a supplement or alternative medicine has recently increased.

Changizi-Ashtiyani et al. in a study examined the effects of barberry root extract on cholesterol, triglycerides, and high and low-density lipoproteins and compared its effects with those of oral atorvastatin. They showed that the ethanol extract of barberry root reduced cholesterol and elevated the levels of triglyceride (TG), high-density lipoprotein (HDL), and low-density lipoprotein LDL. Atorvastatin, too, reduced the amount of cholesterol and triglycerides. Probably one of the alkaloids found in this plant, i.e., berberine, through various mechanisms, affected the process of absorption, synthesis, and excretion of lipids. However, further studies are needed to understand the involved mechanisms (Changizi-Ashtiyani et al. 2013b ▶, Arayne et al., 2007 ▶). Recently, studies have also reported that berberine reduces cholesterol with a mechanism different from statin drugs. If statin and berberine are taken together, they can inhibit cholesterol much better than when they are used alone. Berberine increases the production of a receptor in the liver which bonds with cholesterol and facilitates its excretion (Doggrell et al., 2005 ▶, Rajaian et al., 2002 ▶). Berberine reduces lipid peroxidation effectively by its antioxidant property. Thus, it supports the antioxidant enzymes such as superoxide dismutase (SOD) against oxygen free radicals and hydrogen peroxide (Ziai et al., 2010 ▶, Thirupurasundari et al., 2009 ▶). Antioxidants prevent high blood cholesterol and possibly increase excretion through cholesterol release and consequently reduce its value. In addition, alkaloids inhibit the synthesis of cholesterol (Matralis et al., 2013 ▶). Since barberry also contains powerful antioxidants (Mokhber-Dezfuli et al., 2014 ▶, Motalleb et al., 2008 ▶), its effect on fat loss is not unexpected. The inhibitory effects of barberry root extract on oxidative stress have also been reported (Eshraghi et al., 2011 ▶). Doggrell and colleagues also showed that berberine significantly reduced cholesterol (Doggrell et al., 2005 ▶). Shidfar and colleagues indicated that the administration of aqueous extract of the grainy fruit of mountain barberry (*Berberis harrisoniana*) had an important role in reducing blood cholesterol and triglyceride (Shidfar et al., 2012 ▶).

Clinical trial studies suggest that the aqueous extract of barberry is highly able to efficiently increase cardiac contractility, and reduce blood pressure by reducing peripheral resistance. Berberine increases mRNA activity and increases the expression of LDL-C receptors, which in turn improve the plasma clearance through receptor-mediated endocytosis, which consequently leads to the inhibition of cholesterol biosynthesis of the cell. Hence, among the organic medicinal herbs, berberine may be an ideal candidate for the treatment of hypercholesterolemia. Berberine has beneficial effects on inflammatory fever because of the presence of citric acid, malic acid, and ascorbic acid in its aqueous extract. The aqueous extract of barberry has a great potential as future green medicine (Pradhan et al., 2013 ▶).

Studies have shown that berberine reduces the density of serum cholesterol, triglycerides, and LDL-C in patients and animal models with dyslipidemia. Berberine can act as an AMPK (5'adenosine monophosphate-activated protein kinase) and increases AMP/ATP and is able to inhibit the biosynthesis of ATP in mitochondria. Berberine actually increases insulin sensitivity and is capable of inhibiting alpha glucosidase and adipogenesis and thus acts as an anti-obesity agent. Furthermore, berberine can increase LDL receptor mRNA and as an antioxidant can scavenge free radicals (Shidfar et al., 2012 ▶).

**The effects of barberry extract on liver function tests**

Taheri and colleagues evaluated the effects of root extract of *Berberis vulgaris *and atorvastatin on the levels of liver enzymes in hyper cholesterolaemiac male rats and showed that the enzymes alanine aminotransferase (ALT) and alkaline phosphatase (ALP) increased in the control group receiving only fatty food. While these levels decreased in both the experimental groups receiving the extract of barberry and in the group receiving atorvastatin, no significant changes were observed in aspartate aminotransferase (AST). Therefore, due to the antioxidant properties of the extract and its effects on the activity of liver enzymes, it could be suggested that the plant extracts could improve the function of liver enzymes.

Polyphenolic compounds are important antioxidants. These compounds, especially flavonoids, also have a protective effect on the liver against damage caused by free radicals and toxins in the liver. The inhibitory effects of barberry root extract on oxidative stress have been recently reported (Taheri et al., 2012 ▶). Eshraqi also investigated the antioxidant effects of Jujube plants, barberry, purslane and acanthus on liver oxidative system, red blood cells, and hemoglobin non-enzymatic glycosylation and showed that glycosylation of hemoglobin in the presence of the studied plants was well prohibited, most effectively by acanthus and barberry. Lipid peroxidation was well controlled in the presence of various concentrations of purslane, barberry, and acanthus. These plants in some concentrations showed antioxidant effects, too (Eshraghi et al., 2011 ▶). Studies also showed that the aqueous extract of mountain barberry fruit could activate liver function and was effective in lipid analysis and possibly in adjusting their levels in blood (Rajaian et al., 2002 ▶).

Berberine which is an organic cation simply passes through cell membrane and forms a complex with DNA molecule, causing structural changes in DNA resulting in the imposition of regulatory functions of the genes and disruption of the differentiation process (Yin J et al., 2008a ▶, Yuan Heng et al., 1961 ▶). In addition, while fetal cells are dividing, the formation of proto berberine complexes with DNA can stop the growth of the fetus. The reduction of maternal and fetal blood lipids and blood glucose can also be effective in reducing the growth of the fetus. Berberine can improve liver function and bile secretion and reduce LDL (Yin et al., 2008b ▶, Wu LY et al., 2009 ▶). Berberine similar to metformin is effective in lowering blood sugar and the mechanisms in both involve inhibiting aldose reductase, inducing glycolysis, and preventing resistance to insulin by increasing insulin receptor expression. (Yin et al., 2008b ▶, Lou et al.,2011 ▶, JU et al.,1990 ▶). 

Hermenean et al. studied the hepato protective properties of *Berberis vulgaris *extract on carbon tetrachloride-induced hepato toxicity in rats. The results showed that carbon tetrachloride administered in rats could alone increase liver enzyme levels and in histological studies extensive necrosis and fibrosis were observed in liver. However, following the administration of barberry extract with carbon tetrachloride, the ALT, AST, gamma glutamyl transferase, direct bilirubin, total protein, and malondialdehyde (MDA) reduced and the amount of glutathione (GSH), superoxide dismutase (SOD), catalase (CAT), and glutathione peroxidase (GPx) increased. Thus, according to the results of the majority of the studies, this extract has hepato protective effects due to its antioxidant properties (Hermenean et al., 2012 ▶).The study done by Abd El-Wahab et al. in 2013 ▶ showed the impact of the raw extract of barberry and its effective alkaloid (barberine) on the suppression of lipid peroxidation in liver cells which suggested a promising compound in the treatment of hepatic oxidative stress (Abd El-Wahab et al., 2103 ▶).

The results of a study by Hanachi and colleagues on the effects of the aqueous extract of barberry with the concentrations of 50, 100, and 250 mg/kg by gavage for 7 weeks on the process of apoptosis in rats with liver cancer showed that in a dose-dependent process it could promote apoptosis (Hanachi et al., 2008 ▶).

Falah-Huseini et al. in a study on the intra peritoneal injection of hydro alcoholic barberry root extract at a doses of 300, 600, and 900 mg/kg in rats with liver injury induced by CCL_4 _showed that the extract at doses 30 times more than the usual dose improved liver damage (Falah-Huseini et al.,2010 ▶). The antioxidant, cyto protective, and hepato protective properties of barberry have been reported in several experimental studies, too (Abd El-Wahab et al., 2103 ▶, Eshraghi et al., 2011 ▶).

According to most studies, the majority of the medicinal properties of the plant are due to the various alkaloids present in different parts of it. The fruit contains dihydro palmitinium hydroxide which is an anti-estrogen substance that causes endometrial atrophy and crumple of gastric glands. These changes result in nutrition disorders in the fetus and its growth. Barberry root is a laxative which opens hepatic duct and cystic duct (Javadzadeh et al., 2012 ▶).

Several studies on the antimicrobial and antioxidant effects of the aqueous extract of barberry showed that the fruit could act both as an antioxidant (Eshraghi et al., 2011 ▶) and a natural anti-bacterial agent (Dashti et al., 2014 ▶).

Studies showed that barberry had numerous health benefits, including hepato protective and hypoglycemic ones and can be used as a medicinal herb to treat a variety of injuries, such as diabetes, liver disease, gallbladder pain, digestive, urinary tract diseases, and gallstones. Several animal studies indicated that the aqueous and alcoholic extracts of the plant fruit had antihypertensive properties (Saleem et al., 2005 ▶, and Meliani et al., 2011 ▶).

**The anti-inflammatory and antioxidant effects of barberry extract**

In a series of studies, the barbamine present in barberry was mentioned as an anti-inflammatory and antioxidant agent (Eshraghi et al., 2011 ▶, JU et al., 1990 ▶). It is also shown that the antioxidant effect of barberry on hepatocytes is similar to those of silymarin which is a known hepato protective agent (Tsai et al., 2004 ▶).According to a study, the activities of antioxidant enzymes such as catalase and superoxide dismutase in the livers of the rats with barberry plant in their diets were higher than in the control group, implying the barberry's inhibitory effect on lipid peroxidation through increasing the antioxidant enzymes (Murugesh et al., 2005 ▶). In a study in 2009, it was shown that the barberry plant had positive effects on the liver of diabetic rats and might be effective in preventing complications of diabetes as it regulated glucose homeostasis by decreasing glucose production and oxidative stress (Singh J et al., 2009 ▶). In the study by Lee et al. (2006) ▶ it was shown that the berberine in barberry could lower lipogenesis and had its inhibitory effects on lipid peroxidation (Lee et al., 2006 ▶). Therefore, we conclude that it is possible to use barberry as antioxidant supplements in illnesses such as diabetes, liver disease, and atherosclerosis as prevention or treatment (Eshraghi et al., 2011 ▶).

In an in vitro experiment in which human cell lines were used, it was shown that berberine could inhibit activator protein1 (AP-1) as an important agent causing inflammation and cancer. Similarly, in a study using human peripheral lymphocytes, it was proved that berberine was able to inhibit lymphocyte transformation which would justify its anti-inflammatory properties as it could inhibit DNA synthesis in active lymphocytes. According to another study, in case of tissue injury, berberine had an influential direct role in many stages of platelet-dependent inflammatory process. Here, berberine showed dose-dependent inhibition of arachidonic acid release from cell membrane phospholipids, inhibition of thromboxane A_2_ from platelets, and inhibition of thrombus formation (Fukuda, et al., 1999 ▶).

Ebrahimi-Mameghani and colleagues in their study on the effects of *Berberis vulgaris *extract on blood pressure and inflammatory markers showed that black barberry had no effects on serum concentrations of interleukin-6 (IL-6) and C-reactive protein (CRP). The results of this study showed no effects on systolic and diastolic blood pressure and inflammatory markers by processed black barberry and the positive effect of apple vinegar on interleukin-6 (Ebrahimi-Mamaghani et al., 2009 ▶).

Shamsa and colleagues studied anti-inflammatory effects of barberry fruit on guinea pigs and showed that the addition of black barberry fruit extract to the isolated terminal ileum of guinea pigs, similar to dexchlorpheniramine caused a decrease in histamine dose-dependently and like atropine it decreased acetylcholine in a dose-dependent manner. Histamine causes the release of IL-6 stored in T-cells. Histamine also causes dilation of local blood vessels and increases capillary permeability and leakage of large amounts of fluid into the interstitial spaces and inflammation. Thus, it is likely that the use of black barberry fruit extract can lower IL-6 concentration through decreasing histamine and consequently can help reduce inflammation (Shamsa et al., 1999 ▶).

In a study by Kiasalari and colleagues on the effect of *Berberis vulgaris *extract on acute inflammation (caused by plantar injection of formalin, xylene to ear and acetic acid to peritoneum) and chronic one (caused by gauze implanted in the groin) in rats, it was shown that the consumption of alcoholic extract of the plant fruit was able to reduce acute and chronic inflammation (Kiasalari et al., 2011 ▶). Minaiyan et al. in a comparative study of the effects of fruit extract of barberry, berberine chloride, and corticosteroids on a rat model of ulcerative colitis concluded that the gavage of the extract that was virtually free from berberine was significantly effective in preventing colitis injuries which could be due to its anthocyanin compounds (Minaiyan et al., 2011 ▶).

The phyto chemical analysis of barberry root and stem revealed the presence of proto berberines and bisenzyliso quinoline alkaloids (berbamine, tetrandrine and chondocurine) with anti-inflammatory and immunosuppressive properties (Li et al., 1989 ▶). The results of a comparative study done by Minaiyan et al. on the effects of barberry fruit extract and chloride berberine on the rats' acetic acid-induced colitis showed that the oral administration of the extract at a dose of 750-1500 mg/kg was useful against the colic. 

They concluded that since fruit extract is nearly devoid of berberine so this protective effect may be attributed to the anthocyanin compounds present in the plant (Minaiyan et al., 2011 ▶).

The major sugars of fruit are glucose 8.48 g/dl and fructose 6.12 g/dl and the organic acids are malic and citric. The main antioxidant capacity of barberry lies in its high levels of phenols and flavonoids such as anthocyanins and other polyphenolic compounds which account for its unique place among all fruits (Ozgen et al.,2012 ▶).

**The effects of barberry extract on blood sugar**

Diabetes mellitus is a complex metabolic disorder caused by a deficiency or lack of insulin secretion or reduced insulin sensitivity of tissues (Ahangarpour et al., 2012 ▶). About 800 kinds of medicinal plants have been used in traditional medicine to treat diabetes. The hypoglycemic effects of many of these plants in animal models and clinical studies have been studied and approved (Arumugam et al., 2013 ▶; Ahangarpour et al., 2012 ▶).

Having given the history of barberry plants used in traditional medicine for lowering blood sugar, Ahangpour et al. examined the effects of barberry extract on insulin release from the isolated pancreatic islets of male mouse. The findings of this research indicated an increase in insulin secretion in the presence of the extract. Thus, it may be suggested that one blood sugar lowering mechanism by barberry is through its effect on pancreatic islets. It is noteworthy that the increasing effect of the extract on insulin secretion is less than that of glibenclamide (Ahangarpour et al.,2012 ▶).

In a study conducted by Yin and colleagues it was found that berberine had a lowering effect on glucose in hepatocytes (Yin et al.,2002 ▶).Moreover, in another study, it was proved that the berberine in barberry acted similar to metformin in increasing insulin sensitivity in rats receiving high fat diets (Gao et al.,1997 ▶).Berberine can act as an activated protein kinase (AMPK) and can increase the ratio of AMP to ATP and inhibit the biosynthesis of ATP in mitochondria (Yin et al., 2008b ▶;Shidfar et al., 2012 ▶). In addition to increasing insulin sensitivity, berberine acts as an alpha-glucosidase inhibitor (Tang et al., 2006 ▶; Leng et al., 2005 ▶) and inhibits adipogenesis, hence, exhibits anti-obesity activity (Kim et al., 2009 ▶). Berberine also increases LDL-C receptors and as an antioxidant can scavenge free radicals (Tomosaka et al., 2008 ▶; Shidfar et al., 2012 ▶).

Meliani and colleagues also studied the effects of *Berberis vulgaris *extract on normal and diabetic rats, and showed that the administration of saponin and barberry extract for a week reduced blood sugar in normal rats. Furthermore, in addition to lowering blood glucose in diabetic rats, it lowered cholesterol and triglycerides as well. Therefore, the plant can be effective in the treatment of diabetes (Meliani et al., 2011 ▶).

In another study, Ashraf and colleagues examined the effect of aqueous extract of Zarafshan barberry root on testes and testosterone levels in streptozotocin-induced diabetic rats. In this study, diabetes caused a significant decrease in testosterone levels, diameter of seminiferous tubules, spermiogenesis index, and the thickness of the epithelium as well as a significant increase in the thickness of the interstitial tissues and blood glucose levels in diabetic group in comparison to the other groups. By using barberry root, these changes were brought to normal levels (Ashraf et al., 2013 ▶).

Abd El-Wahab and colleagues (2013) ▶ in an in vitro study examined the antioxidant, anti-acetyl cholinesterase, anti-cancerous, and anti diabetic properties of *Berberis vulgaris *and its active compound, i.e., berberine. The study showed that the plant compounds had the biological potential of inhibiting lipid peroxidation (Abd El-Wahab et al., 2013 ▶).

The results of the study done by Shidfaret al. showed that a daily intake of 3 g fruit extract over a period of 3 months had beneficial effects on the lipoprotein and apoprotein concentrations as well as triglyceride and glycemic control in patients with type 2 diabetes (Shidfar et al., 2012 ▶).

Studies have shown that the activity of berberine is comparable to sulfonylureas and metformin. The administration of berberine can reduce FBS (fasting blood sugar) and HbA_1_C in patients with newly diagnosed type 2 diabetes. Berberine reduces weight, increases insulin sensitivity, decreases insulin resistance, and thereby reduces blood sugar in genetic models of type 2 diabetes (Shidfaret al., 2012 ▶).

The administration of barberry root aqueous extract in streptozotocin-induced diabetic rats showed a significant anti hyperglycaemic effects. This suggests that the effects may be related to the presence of saponin in the extract with its stimulatory effects on residual beta-cell (Meliani et al., 2011 ▶).

**The effects of barberry extract on thyroid function**

The thyroid gland, secreting thyroxine (T_4_) and triiodothyronine (T_3_) hormones, has undeniably profound effects on metabolism. Researchers found that blood lipid levels were inversely correlated with thyroid hormone levels and by increasing hormones, the lipid profile levels dropped.

Even in patients with hypothyroidism, the level of LDL cholesterol increases while it is reduced in hyperthyroidism (Zarei et al., 2012a ▶). Studies also show that levels of blood fats such as cholesterol and triglyceride (TG) increase in groups with high fat diet. There is also a direct correlation between fat and leptin, while the correlation between T3 and leptin is an inversely significant one. However, blood lipid levels do not correlate with TSH levels. This actually represents an association between fat, leptin, and thyroid hormones (ShekarForosh et al., 2012 ▶; Zarei et al., 2013b). Accordingly, Zarei et al. in a study surveyed the effects of the root extract of barberry and atorvastatin on thyroid hormone levels in rats with hypercholesterolemia. The results showed that in the groups receiving the extract of the barberry roots and atorvastatin the levels of thyroid hormones (T_3_ and T_4_) increased while the level of thyroid-stimulating hormone (TSH) decreased in the groups receiving atorvastatin. 

The groups treated with the extract of barberry root did not show significant changes in TSH levels. The increased levels of thyroid hormone and decreased TSH levels in the group receiving atorvastatin were possibly due to negative feedback effect of thyroid hormones on TSH. The increased levels of T3 andT4 with no effects on TSH levels in the experimental groups receiving barberry root extract indicated euthyroid hyper thyroxinemia.

These changes might be caused by increased plasma proteins, including albumin, reduced fat and leptin levels, and increased neuropeptide Y as well as stimulated hypothalamic para venericular nuclei which were possibly made by alkaloid compounds found in the plant. It seems that the extract at above-mentioned doses caused pathological changes in the pituitary-thyroid axis (Zarei et al., 2015 ▶).

Due to multiple properties of the root and fruit of barberry on controlling the complications of diabetes, reducing and controlling lipid profiles and liver enzymes, and its obvious antioxidant and anti-inflammatory properties, as well as its ability to influence the secretion of the thyroid hormones, therefore this plant can be considered as a drug candidate for the prevention and control of cardiovascular diseases, diabetes, thyroid disorders and liver disease.
